# Promoting Health Equity Through the Power of Place, Perspective, and Partnership

**DOI:** 10.5888/pcd20.230160

**Published:** 2023-07-27

**Authors:** Derek M. Griffith, Dawn Satterfield, Keon L. Gilbert

**Affiliations:** 1 Racial Justice Institute, Center for Men’s Health Equity, Georgetown University, Washington, DC; 2 Division of Diabetes Translation, National Center for Chronic Disease Prevention and Health Promotion, Centers for Disease Control and Prevention, Atlanta, Georgia; 3 Brookings Institution, Washington, DC; 4 Department of Behavioral Science and Health Education, Institute for Healing Justice and Equity, College for Public Health and Social Justice, Saint Louis University, Saint Louis, Missouri

## Abstract

The 10 articles in the *Preventing Chronic Disease* (PCD) special collection on health equity highlight that a commitment to self-reflection, cultural humility, and lifelong learning are foundations of health equity science and that the field is interdependent with the perspectives and context of communities.

Three themes — place, perspective, and partnership — emerged from the PCD special collection. The articles embody the principles outlined in the Healthy People definition of health equity and CDC’s CORE Health Equity Science and Intervention Strategy. They highlight the critical role that context, qualitative methods, and community-based participatory research play in efforts to achieve health equity. However, the science of achieving health equity is rooted in antiracism principles; the “inner work” of learning, unlearning, relearning, and co-learning; and the efforts to equip communities to act, research, and intervene for themselves. Without these added critical structural lenses, health equity science will continue to fail to achieve its goal.

**Summary**

**What is known on this topic?**

Health equity is an almost universal priority, yet the goals, objectives, plans, and resources required to achieve health equity remain unclear.

**What is added by this report?**

The concept of a “wicked problem” is a useful way to note how achieving health equity differs from other public health goals and objectives.

**What are the implications for public health practice?**

While there is a tendency to focus on programs and policies, the fundamental work of health equity is in the learning, unlearning, relearning, and co-learning of public health professionals, communities, and community-based participatory research partnerships.

## Introduction

Fifty years ago, Rittel and Webber (1) coined the term “wicked problem” to describe scientific problems for which the root causes and the path for resolving problems are not clear. Wicked problems are those that do not have a definitive formulation or solution. Considered to be a symptom of another problem, wicked problems are particularly challenging because interested parties differ in the values and interests they apply to resolving them (1). Achieving health equity is complicated and can be viewed as a uniquely wicked problem because of the web of historic, geographic, economic, social, structural, political, commercial, and other health determinants that intersect dynamically, bundling even more thickly when newer threats impinge on hopes for health equity (eg, public health infectious disease emergencies, climate-related disasters). Achieving health equity is further complicated by the challenge of effectively communicating to decision makers the logic, status, and depth of the problem itself (1).

Public health struggles to conceptualize, define, and operationalize a cohesive plan to achieve health equity almost 40 years after the Heckler Report (2). The report documented inequities in key health indicators among demographic groups of the US population and launched a new generation of health disparities research and practice. Thus, despite the volume of resources committed to this goal and robust acknowledgment that health equity is important, differences persist in perspectives on the goals, objectives, plans, and resources required to achieve health equity — a state where everyone has a fair and just opportunity to attain their highest level of health (3–5).

The information needed to understand and pursue health equity are integrally intertwined, limiting the ability to characterize and define the problem in a way that enables a solution (1). Most health equity research has not grappled with this penultimate goal but has focused on identifying causal associations that describe health inequities instead of interventions that employ antiracism principles and move the nation toward health equity (6). Interventions and efforts to achieve health equity that have been tested were limited by resource, time, and other considerations external to the problem.

The Call for Papers for this special collection of *Preventing Chronic Disease* (PCD) on health equity concluded, “Health is not just the absence of disease but also the presence of resources and supports that people need to thrive.” The collection of papers herein embodies the theme, “Health Equity in Action: Research, Evaluation, Policy,” and builds on the Healthy People 2020 roadmap for health equity. This PCD collection also reflects the 3 overarching goals of Healthy People 2030: 1) “eliminate health disparities, achieve health equity, and attain health literacy to improve the health and well-being of all,” 2) “create social, physical, and economic environments that promote attaining the full potential for health and well-being for all,” and 3) “engage leadership, key constituents, and the public across multiple sectors to take action and design policies that improve the health and well-being of all” (7). In 2021, the Centers for Disease Control and Prevention (CDC) launched an agency-wide strategy to holistically reimagine their approach to health equity aligned with these goals. The agency committed to integrating health equity in all aspects of what they do (3,5) by outlining CDC’s CORE Health Equity Science and Intervention Strategy to “**C**ultivate comprehensive health equity science, **O**ptimize interventions, **R**einforce and expand robust partnerships, and **E**nhance capacity and workforce engagement” (3,5).

## Review of Articles in the Special Collection

The 10 articles that comprise this PCD special collection on health equity exemplify the principles outlined in the Healthy People definition of health equity and CDC’s CORE Health Equity Science and Intervention Strategy. All the articles acknowledge social determinants of health inequities in their introductions, often attending to the PCD call for deep, rather than superficial, descriptions of this phenomenon. In reviewing the articles, 3 themes emerged: place, perspective, and partnership. Through demonstrating the roles that race-based residential segregation, food deserts, neighborhood conditions, loss of lands, and other built environmental factors play, literature on health inequities has consistently demonstrated that the “place” where people live, work, play, and engage in spiritual and religious practice has implications for their health. Although some articles in this collection focus on the importance of quantitative methods, the second theme that emerged highlighted the importance of effective communications and the strengths of qualitative research (8). Qualitative research provides insight with an “insider’s view” on injustices and the hope for action to improve people’s health and well-being (8). The third theme to emerge was the critical role of engagement with community partners (9). In the remainder of this section, we review the groups of articles that are consistent with each of these 3 themes.

### Place: the importance of geographic context

Using census tract–level rates of cardiovascular morbidity and mortality for Black residents in metropolitan Atlanta, Georgia, Kim and colleagues (10) identified 106 resilient neighborhoods and 121 “at-risk” neighborhoods where Black residents had substantially lower-than-expected and higher-than-expected rates of cardiovascular disease events, respectively, despite similarities in their neighborhood income levels. Smiley and colleagues (11) analyzed secondary quantitative data in Los Angeles, California, to understand whether the racial composition of neighborhoods is associated with exposure to menthol cigarette marketing. The highest level of exposure to marketing was in African American neighborhoods, compared with neighborhoods composed of residents from other racial and ethnic groups (11). Coats and colleagues (12) examined how race, ethnicity, and gender intersect to affect employment loss and food insecurity in St. Louis, Missouri. Cardarelli and colleagues (13) conducted focus groups in Martin County, Kentucky, that explored perceptions of the local food environment and assessed the potential acceptability of an intervention strategy to promote equity in obesity prevention in this rural Appalachian community.

### Perspective: the importance of effective communication and qualitative research

Brian and Weintraub (14) remind us that prevention is a cornerstone of public health practice. Efforts to integrate dental programs within clinical care that focus on prevention, screening, and risk assessment could improve physical and mental health outcomes and help to prevent chronic diseases. Oral health care should be a public health priority, including in the response to the COVID-19 pandemic. Brian and Weintraub (14) argue that the introduction of unique barriers to reopening dental practices disproportionately affected populations at high risk for contracting and suffering serious complications and death from the virus.

Calanan and colleagues (15) described the 2-phase development of the *Health Equity Guiding Principles for Inclusive Communication *(*Guiding Principles*) (16). The first phase created a tool to guide the development of scientific and other communications. The *COVID-19 Health Equity Style Guide* provided guidelines for preferred terminology and other best practices from communication science literature and subject matter experts; then, the guide was shared informally with other CDC staff not directly involved in the COVID-19 response. The second phase created a public-facing resource available for all public health practitioners and partners to apply an equity-centered approach to communicate information to improve the health of all communities respectfully. The *Guiding Principles* website covers 2 broad considerations for developing respectful, inclusive, and nonstigmatizing communications: 1) to understand and frame information in terms of social and health inequities, and 2) to apply the best culturally responsive practice for the intended audience through language use, image selection, and other guidelines. The *Guiding Principles* serve as a starting point and an approach that is not a mandate but rather an important resource that has been presented to a range of partners as public health practitioners and partners consider how to adopt these guidelines in all types of communication (15).

In addition to these perspectives of public health professionals, 3 articles highlight the importance of qualitative research (17,18). Qualitative inquiry helps to explore phenomena in context, including the natural settings of “place,” and it elevates the voices of those who experience disproportionately poor health (18). As is evident from the article by Cardarelli and colleagues (13), qualitative methods often serve as a primary source for explaining how and why inequities exist and what may work to promote equity in their communities (18). Felner and Henderson (18) present and respond to the need for additional pragmatic guidance on thoughtfully designing and conducting a robust qualitative data analytic strategy to produce findings that have implications for advancing health equity. Also, to facilitate self-reflection, Felner and Henderson (18) recommend that researchers undertake reflective “memo writing” on what they are learning, including exploring their biases.

Satterfield et al (17) elicit the perspectives of children, parents, and educators to explain the sustained appeal of Eagle Books, a series of 4 books to educate young American Indian and Alaska Native children about type 2 diabetes and related conditions. Tribal leaders guided CDC and the Indian Health Service in the development of the books to use traditional ways of teaching children how to stay healthy. The voices of volunteers participating in the qualitative evaluation allowed the researchers to identify critical themes that help explain the interest in the stories over time. A major theme from their findings was that children identified with the characters who “look like me” and with cultural values such as generosity and caring for one another. Several educators and parents shared stories of children who championed food and activity messages for their families and friends. The authors cite quantitative studies by independent Eagle Books programs that documented significant intentions to make healthier food and activity choices after exposure to the stories (17).

### Partnership: the power of community engagement

Two articles illustrate the principles of community-based participatory research (CBPR), inviting the direct, equitable participation of people with relevant lived experience in all aspects of research and application of benefits (9,19,20). Ellis et al (21) argue that family-focused interventions to facilitate chronic disease management should center on racial health equity and explicitly consider family health history, sociocultural and contextual factors, and community-engaged participatory approaches to work “inside, outside, and alongside” families. They contend that deeper attention to the family relationship context for chronic disease management is essential to improving outcomes among adults who are disproportionally affected by chronic diseases (21). They recommend a framework for disciplinary self-critiques that can help examine how racism has hampered efforts to achieve health equity.

Akintobi et al (22) describe how their Prevention Research Center (PRC) relied on community wisdom and the governance of a long-established community coalition board. They described how community members taught the PRC that some terms used in COVID-19 media messaging fostered anxiety and mistrust of public health and health care systems. The community coalition board facilitated public health disaster health literacy to refine messaging about mitigating the virus to be more congruent with framing that resonates with the community. The community coalition board also prioritized patient-centered models of integrated mental health care within primary health care. They described how they learned of the toll of pandemic stressors that adversely affected mental health and recommend that public health practitioners understand and communicate the complexities of health disparities in the context of historical and current social determinants of health.

## Implications for Public Health

Across this PCD health equity special collection’s themes of place, perspective, and partnership, the role of the context and focus of our public health interventions is worth noting. Since opportunities to be healthy are shaped by people’s daily environments, “place” is a critical setting for health equity science. Incorporating the characteristics of the environment provides opportunities for public health practitioners to locate their work with communities in a particular setting and to consider other social and structural determinants of health. While it is crucial to create and widely adopt behavioral practices that promote health and well-being, public health professionals recognize the unique role of place for optimizing intervention opportunities that can yield the healthiest behavioral and health outcomes (3,5).

The second theme of perspective highlights the importance of communications and qualitative research. Considered 1 of the 10 essential public health services (23,24), effectively communicating in inclusive and supportive ways is crucial (16). In addition, although quantitative research is a foundation of epidemiology and other aspects of public health, qualitative research reveals the meaning of experiences and views of participants in the context of their lives and settings (8). Qualitative findings can help identify community assets, explicit and implicit theories, and factors that affect health across levels of the social ecological framework (8).

As reflected in CDC’s CORE Strategies and in arguments made by CBPR scholars for decades, improving local conditions to mitigate the implications of structural racism on health requires meaningful collaboration and work with community organizations. Building on recommendations from Ellis and colleagues (21), pursuits of health equity are bolstered when organizations and institutions share and co-create plans to acquire, mobilize, and utilize resources to work and walk “inside, outside, and alongside” communities. Creating structures and the capacity for researchers, practitioners, and communities to work in partnership is integral to improving understanding of public health problems and creating innovative strategies to solve them. Thus, a primary goal of health equity science is to increase the knowledge, skills, confidence, and motivation to fulfill one’s public health role in ways consistent with the penultimate goal of achieving health equity. The efficacy to achieve health equity is not limited to public health researchers or even their agency’s goals; this quality lies at the heart of community-based partnerships with academic, nonprofit, and local, state, and federal organizations built by residents dedicated to improving health outcomes for their people.

## Recommendations: Toward Fundamental Principles of Health Equity Science

Antiracism provides a vision, framework, and tools to guide efforts to achieve health equity (25). Consistent with antiracism principles (19,25) and the notion of cultural humility (26), this PCD issue highlights the critical role that commitment to self-reflection, self-awareness, and redressing imbalances and injustices plays in helping to change the world to improve the odds that people can be healthy and achieve health equity (25). As we connect this PCD special collection with the larger body of literature, we offer 6 recommendations to guide health equity science. First, health equity research and practice are inclusive of the “inner work” of learning, unlearning, relearning, and co-learning and may not be reduced to the “outer work” of policies, programs, and practices to avoid unhealthy outcomes (27). Second, a significant part of the outer work and inner work reflects the cultural humility and critical awareness and commitment to redressing imbalances needed to achieve health equity. Third, while partnership may be an essential tool in the health equity science toolbox, CBPR is only one approach that communities, researchers, and practitioners may use to inform and guide their collaborative work. Regardless of the approach, it is critical for community, researcher, and practitioner partnerships to include tools and processes to evaluate the effectiveness of their partnership and the implications of their collaborative work for policy and practice. If the goal is to achieve health equity, it is critical to integrate CBPR and other partnership approaches with antiracism principles (19,20,25). A commitment to partnering with communities throughout the research process includes the recognition of racism as a public health problem (28) and a fundamental determinant of health inequities. A commitment to addressing racism in the partnership or mitigating and undoing racism should ensue as part of the work (19,25). Future iterations of CBPR principles should be revised to more explicitly integrate antiracism principles (19,25) and community priorities (20,29). Fourth, as the CDC CORE Strategy outlines, efforts to achieve equity should seek to enhance, or increase, the capacity of community members to define their own etiology of health problems and possible solutions (9). A critical aspect of public health professionals’ work is increasing the capacity to communicate in respectful, inclusive, and nonstigmatizing ways (15,16). Building and respecting this type of community power (29) is not only fundamental to CBPR approaches to research, but also helps to create a sustained foundation once achieved. Four decades of health equity research have shown how critical it is for efforts to achieve equity not to be perpetually dependent on external partners. One of the goals of health equity science should be to equip communities to act, research, and intervene as equal partners with academic and public health partners and for themselves. Fifth, the ability to communicate meaningfully is critical to all communities, particularly those that have persistently borne a disproportionate burden of poor health outcomes. Sixth, and finally, identifying SMART (specific, measurable, acceptable, realistic, and time-bound) objectives for health equity is a critical tool to direct needed resources to see the nation through to the goal of achieving health equity. SMART objectives guide almost all other programmatic, funding, and policy efforts in the US because they provide benchmarks, motivation, and perspective on the resources needed to achieve public health goals (4). Creating SMART health equity objectives will elevate health equity science strategies and initiatives across public health practice, policy, and research to mitigate and undo racism to achieve and sustain equal health outcomes.

## Conclusion

Public health is a tool to change the world (30) and a profession that “works to develop public policies that can change the odds that more people can succeed” (31). However, health inequities persist. People disagree about the trade-offs involved in achieving health equity, the speed with which we seek to reach equitable opportunities and outcomes, and whether achieving health equity is possible given the other structural inequities characterized by the notion of structural racism (25). It is important to remember that health equity is a state that has never existed in the US; thus, health equity science has an opportunity to move beyond changes in terminology to build on and sustain efforts to achieve equity (4). Efforts to achieve equity must be rooted in a culture of commitment and accountability to the principles of fairness and justice — foundational structures that will guide us to our destination (4,29). Not simply a moral imperative, health equity is a necessary requisite to reducing the drain on our health care system, health care providers, overall economy, and collective well-being that is currently mired by inequities (4). Ensuring that the public health community collectively does the inner work necessary to decide what it is willing to do to achieve health equity will be critical.
